# Longitudinal survey of self-reported level of entrustment across the first year of clinical practice

**DOI:** 10.12688/hrbopenres.13487.1

**Published:** 2022-01-20

**Authors:** Paul O'Connor, Sinéad Lydon, Orla Mongan, Dara Byrne

**Affiliations:** 1 General Practice, National University of Ireland, Galway, Galway, Co. Galway, H91 TK33, Ireland; 2 Irish Centre for Applied Patient Safety and Simulation, National University of Ireland, Galway, Galway, Co. Galway, H91 TK33, Ireland; 3 School of Medicine, National University of Ireland, Galway, Galway, Co. Galway, H91 TK33, Ireland

**Keywords:** Entruastable professional activities, competency based education, junior doctor, survey

## Abstract

**Background.** Newly graduated medical students often report that they lack the skills required to care for patients, and feel unprepared for clinical practice. However, little is known about when, and if, they acquire these skills in practice. The aim of this study was to assess self-reported level of entrustment in, and frequency of performance of, the seven Entrustable Professional Activities (EPAs) comprising the EPA framework for interns in Ireland. EPAs describe discrete activities that are essential to a particular profession.

**Methods**. Self-report survey data were collected from doctors in the Republic of Ireland during their first year of clinical practice at four different time points during 2020/21.

**Results**. Response rates to the survey varied from 73.3% (126/172) at Time 1 to 25.6% (44/172) at Time 4. After three months, the respondents reported that they could execute all of the EPAs, inclusive of 12 essential procedural skills, with indirect supervision. As the year progressed there was an increase in the proportion of respondents reporting that they performed the EPAs at least once a week. However, the proportion of respondents performing five of the essential procedural skills (e.g. nasogastric tube insertion) remained low across all time points.

**Conclusion**. Consideration should be given as to how to better prepare medical students to execute these EPAs, how the interns can be better supported during the first quarter of internship. The findings from this research are positive. However, there is an urgent need to carry out formal assessments of entrustability, rather than relying on self-report.

## Introduction

It has consistently been found that junior doctors lack the skills required to care for patients, and feel unprepared for clinical practice ^1, 2^. The training and support received by junior doctors is variable, and dependent on the setting and clinical teams in which they are placed ^3^. Moreover, junior doctors continue to the next stages of training, not based upon a determination of whether they have developed the necessary competencies, but rather as a result of the time they have spent in the role ^4^. The issues associated with a time-based approach to training has led to an interest in a Competency Based Medical Education (CBME) for the training of junior doctors. CBME is concerned with outcomes of the education experience, independent of time spent in education ^5^. A particular approach to CBME that has received interest within the context of the training of junior doctors is Entrustable Professional Activities (EPAs) ^6^.

EPAs are an effort to bridge the gap between the theory of competencies and practical clinical work ^7^. EPAs are units of professional practice that capture essential competencies in which trainees must become proficient ^8^. EPAs describe discrete activities that can be entrusted to trainees, are essential to the profession, and encapsulate one or more core competencies ^9^. EPAs use observable work descriptors (e.g., clerk a patient) as opposed the person-descriptors (e.g., good communicator) ^9^. EPAs provide supervisors with a framework to support the delegation of activities to trainees ^8^. EPAs have potential for improving the efficiency of training of junior doctors, and ensure that they perform only the activities they have been deemed safe to perform ^10^.

In Ireland, a framework of seven EPAs (see Table 1) has been developed for the intern year (the first year of clinical practice after graduation in the Republic of Ireland). This EPA framework was developed through an iterative eight-stage consensus building with key stakeholders (see O’Dowd *et al.* ^4^ for a description of this process). Levels of entrustment of trainees for each EPA are made on a scale of 1 to 5:

**Table 1.** Irish intern year EPAs (adapted from O’Dowd *et al.* ^4^).

EPA	Description
EPA 1: Clerk a patient.	The doctor can clerk a patient in the outpatient and day care setting, admit a patient to the ward and have a good understanding of decision to admit criteria.
EPA 2: Request and interpret basic investigations.	The doctor can request appropriate and interpret basic diagnostic laboratory and radiological investigations.
EPA 3: Perform essential procedural skills.	By the end of internship, the doctor demonstrates confident and is skilled in performing 12 essential clinical procedures: (1) hand hygiene; (2) venepuncture; (3) peripheral intravenous cannulation; (4) blood cultures from a peripheral vein; (5) arterial blood gas sampling; (6) electrocardiogram; (7) nasogastric tube insertion; (8) urinary catheter insertion; (9) preparation, reconstitution, dilution and administration of IV drugs; (10) blood sampling & blood cultures from central line and tunelled line; (11) sterile field set up; and (12) sterile glove application.
EPA 4: Manage the work of in-patient care.	The doctor can manage their daily workload to prioritise, delegate tasks, advance patient flow, and deliver patient centred care.
EPA 5: Prescribe and monitor drugs and fluid.	The doctor can prescribe sagely in compliance with legal requirements in both a hospital and community setting, and in an elective and emergency setting.
EPA 6: Recognise and manage the deteriorating/ acutely unwell patient.	The doctor can identify and respond to the acutely unwell patient appropriately.
EPA 7: Handover and discharge a patient.	The doctor can handover and receive the handover of a clinical case to/from colleagues and manage the discharge of a patient competently.

1. Observation but no execution, even with direct supervision.
2. Execution with direct, proactive supervision.
3. Execution with reactive supervision, i.e., on request and quickly available.
4. Supervision at a distance and/or post hoc (regarded as the threshold for competent independent practice).
5. Supervision provided by the trainee to more junior colleagues ^4^.

The desirable minimum level of entrustability in these EPAs for interns is to be able to execute with reactive supervision (level 3), or supervision at a distance and/or post hoc (level 4). The rationale for the need to reach this level of entrustability is that interns tend to work autonomously with limited support from more senior doctors- particularly during night and weekend shifts ^11, 12^. Lack of supervision has been identified as a contributor to more than half of errors made by interns that ‘played on their mind’ ^13^. This issue of limited supervision is further compounded by the fact that junior doctors are also often unwilling to seek guidance and clinical support from seniors ^12, 14^. Therefore, ideally, interns should be able to execute these EPAs with indirect supervision.

The aim of this study was to assess self-reported level of entrustment within, and frequency of performance of the seven Entrustable Professional Activities (EPAs) comprising the EPA framework for interns in Ireland at four time points in the intern year. This will allow an indication of when, and if, interns are able to execute these EPAs without the need for direct supervision.

## Methods

The study is reported in accordance with the Consensus-Based Checklist for Reporting of Survey Studies (CROSS) ^15^.

### Context

The intern year is the first year of postgraduate clinical practice for doctors in Ireland. Each intern is attached to one of six national intern training networks (ITN) and rotates through four clinical attachments, each of three-month duration, within the hospital group associated with their ITN. This study was carried out in one ITN. This ITN had one main 708-bed hub hospital and 8 smaller affiliated hospitals varying in size from 72 to 359 beds.

### Ethics

Ethical approval was received from the Ethics Board Chairperson of Galway University Hospital (ref CA 2241). All participants provided written informed consent prior to completion of the survey.

### Survey instruments

Two annonymous survey instruments were developed (see *Underlying data* ^16^). In survey A participants were asked to indicate their level of entrustability in each of the seven EPAs in the EPA framework for Irish interns ^4^. For EPA 3 ‘perform essential procedural skills’, participants were asked to indicate their level of entrustability with which they perform the 12 associated procedural skills (see Table 1). The participants provided an entrustability rating from 1 (observation but no execution, even with direct supervision) to 4 (supervision at a distance and/or post hoc). Level 5 was not included as this is level of supervision is not carried out by the interns. Participants were also asked to provide their sex, age, and whether they were a graduate of an Irish or non-Irish medical school. A self-generated identification code was also included (birth date, middle initial, number of older siblings) to track participation. Survey B is the same as Survey A except that the participants were also asked if they perform the EPA/clinical procedure: every shift, every two or three shifts, once a week, less than once a week, or not performed during this rotation.

### Procedure

Interns from one ITN (n=172) were asked to complete Survey A immediately prior to starting internship in May 2020 (Time 1). Interns from the same ITN were asked to complete Survey B in August 2020 (Time 2), November 2020 (Time 3), and May 2021 (Time 4). A link to the online questionnaire was sent by email from the intern coordinator, with a reminder a week later, and a final reminder two weeks later. The participants were also given the opportunity to enter into a draw for an Apple© iPad mini at Times 3 and 4.

### Analysis

The analysis was carried out using IBM SPSS version 22. The data sets of responses for each of the four times the surveys were distributed are available as in the *Underlying data* ^16^. No adjustments were made for missing data.

## Results

### Participants

Table 2 provides and overview of the participants on each of the occasions that the survey was completed. A total of 0.6% of the data was missing.

Table 3 shows the median level of self-reported entrustability for each of the six EPAs and the 12 procedural skills in EPA 3. Table 3 also shows the percentage of participants whose self-reported level of entrustability was level 3 (execution with reactive supervision, i.e., on request and quickly available), or level 4 (supervision at a distance and/or post hoc). This is an important distinction as this means the intern does not believe they require direct supervision in performing these skills.

**Table 2.** Participant characteristics (percentages in brackets).

	Time 1	Time 2	Time 3	Time 4
**Responses** (n=172)	126 (73.3)	91 (52.9)	58 (33.7)	44 (25.6)
**Sex** Female Male	72 (57.1) 54 (42.9)	53 (58.2) 38 (41.8)	36 (62.1) 22 (37.9)	27 (61.4) 17 (38.6)
**Age** 18-24 years 25-34 years >34 years	65 (51.6) 57 (45.2) 4 (3.2)	43 (47.3) 46 (50.5) 2 (2.2)	24 (41.4) 32 (55.2) 2 (34.4)	12 (27.3) 29 (65.9) 3 (6.8)
**Graduate** Irish medical school Non-Irish medical school	123 (98.4) 3 (1.6)	90 (98.8) 1 (1.1)	57 (98.3) 1 (1.7)	44 (100) 0 (0)

**Table 3.** Summary data on self-reported entrustability at the four time points.

EPAs	Median (IR)				% level 3 or 4 entrustability			
	Time 1	Time 2	Time 3	Time 4	Time 1	Time 2	Time 3	Time 4
**EPA 1:** Clerk a patient	2 (1)	4 (1)	4 (1)	4 (1)	46.0	85.6	89.7	93.1
**EPA 2:** Request and interpret basic investigations	3 (1)	4 (1)	4 (1)	4 (0)	57.9	92.3	98.3	97.7
**EPA 3:** Perform essential procedural skills								
1. Hand hygiene	4 (0)	4 (1)	4 (0)	4 (0)	96.0	98.9	100	100
2. Venepuncture	3 (1.75)	4 (0)	4 (0)	4 (0)	75.4	97.8	100	100
3. Peripheral intravenous cannulation	3 (1)	4 (0)	4 (0)	4 (0)	58.7	97.8	100	100
4. Blood cultures from a peripheral vein	2 (1)	4 (0)	4 (0)	4 (0)	48.4	97.8	100	100
5. Arterial blood gas sampling	2 (0)	4 (1)	4 (1)	4 (0)	23.0	78.0	94.8	97.7
6. Electrocardiogram	3 (2)	4 (0)	4 (0)	4 (0)	72.2	95.6	100	100
7. Nasogastric tube insertion	2 (0)	3 (2)	4 (1)	4 (0)	17.5	68.9	87.7	100
8. Urinary catheter insertion	2 (1)	4 (1)	4 (0)	4 (0)	11.9	81.3	96.6	100
9. Preparation, reconstitution, dilution & administration of IV drugs	2 (1)	3 (1)	4 (1)	4 (2)	16.7	41.8	55.2	72.7
10. Blood sampling & blood cultures from central line & tunnelled line	3 (1)	3 (2)	4 (1)	4 (0)	11.1	68.5	82.8	97.7
11. Sterile field set up	3 (1)	4 (0.5)	4 (1)	4 (0)	54.8	97.8	98.2	100
12. Sterile glove application	3(1)	4 (0)	4 (0)	4 (0)	81.6	98.9	100	100
**EPA 4:** Manage the work of in-patient care	3 (1)	4 (0.5)	4 (0)	4 (1)	56.3	96.6	98.3	100
**EPA 5:** Prescribe & monitor drugs and fluid	3 (1)	4 (1)	4 (1)	4 (0)	52.0	95.3	98.2	97.6
**EPA 6:** Recognise and manage the acutely unwell patient	2 (1)	3 (1)	3 (1)	4 (0)	46.8	90.7	94.8	100
**EPA 7:** Handover & discharge	2 (1)	4 (1)	4 (1)	4 (0)	45.2	97.6	100	100

Table 4 shows the percentage of respondents who reported that they perform the six EPAs and the 12 clinical skills in EPA 3 at least once a week during the three time points at which they were working in the hospital.

**Table 4.** Percentage reporting that they perform the EPA and 12 clinical procedures at least once a week at Time 2, 3, and 4.

EPAs	Time 2	Time 3	Time 4
**EPA 1:** Clerk a patient	51.1	62.1	63.6
**EPA 2:** Request and interpret basic investigations	97.8	100	100
**EPA 3:** Perform essential procedural skills			
1. Hand hygiene	98.9	100	100
2. Venepuncture	97.8	98.3	97.7
3. Peripheral intravenous cannulation	98.9	100	95.5
4. Blood cultures from a peripheral vein	65.9	70.7	88.6
5. Arterial blood gas sampling	28.6	41.4	56.8
6. Electrocardiogram	60.4	75.9	81.8
7. Nasogastric tube insertion	7.7	6.9	29.5
8. Urinary catheter insertion	14.3	19.0	31.8
9. Preparation, reconstitution, dilution & administration of IV drugs	13.5	8.6	11.4
10. Blood sampling & blood cultures from central line & tunnelled line	13.3	12.1	25.0
11. Sterile field set up	36.7	37.9	31.8
12. Sterile glove application	52.2	44.8	50.0
**EPA 4:** Manage the work of in-patient care	97.7	96.6	100
**EPA 5:** Prescribe & monitor drugs and fluid	97.7	100	95.2
**EPA 6:** Recognise and manage the acutely unwell patient	77.9	74.1	88.1
**EPA 7:** Handover & discharge	94.1	96.6	97.6

The Spearman’s Rho correlation between self-reported entrustability and frequency of performance for all of the EPAs across all three time points (expect for EPA2) was 0.33 (p<.01). The Spearman’s Rho correlation between self-reported entrustability and frequency of performance for the 12 clinical skills across all three time points was 0.42 (p<.01). These can be considered to be moderate correlations.

## Discussion

Medical training has traditionally comprised a time-based apprenticeship model both in the Republic of Ireland as well as internationally. As such, doctors advance to the next stages of training based on time, and not competence. EPAs have been promoted as an approach to support competency-based assessments ^4– 6, 9, 10^ and improve patient safety. The purpose of our study was to assess self-reported level of entrustment within, and frequency of performance of, the seven Entrustable Professional Activities (EPAs) comprising the EPA framework for interns in Ireland in order to identify when, and if, interns are able to, execute them without the need for direct supervision.

Prior to starting internship, the respondents reported a need for direct supervision to carry out many of the EPAs, and the more advanced procedures in EPA 3. This finding is consistent with other studies that have found that high percentages of newly graduated medical students report feeling under-prepared to begin working in a hospital ^11^, and variability in their confidence to perform specific clinical skills ^1^. A national survey of junior doctors in Ireland found that only 51% believed that medical school prepared them well for their intern year ^17^. However, even after only three months of clinical practice there was a large change in in the self-reported level of entrustability, with a much greater proportion of interns reporting that they could complete many of the EPAs and clinical skills with either reactive supervision, or supervision at a distance as compared to the baseline assessment. This is certainly a positive finding, and suggests that internship is effective in increasing the level of competence of junior doctors. However, there may be a cost to patients in terms of compromising safety, the efficiency of the health service (e.g. the need to repeat procedures ^18^, ordering unnecessary tests ^19^) as well as to the interns themselves (e.g. stress or burnout ^13^) in terms of this largely on-the-job approach to skill development. These costs are worth considering, and may help to justify the resources required to establish a competency-based approach to education and training. There is also need to consider what level of entrustability is required for newly graduated medical students as this has implications for by the training and supervision required by interns. This is an issue that has been addressed by the UK General Medical Council for practical procedures and skills ^20^. It is suggested that this should also be considered within the broader context of the EPAs.

The interns reported carrying out the majority of the EPAs and clinical procedures at least once a week, with an increase in the frequency of performance during the year. It could be postulated that as interns complete the EPAs and clinical procedures more, they then become more confident in their ability- as indicated by the moderate correlation between self-reported entrustability and frequency of performance. However, there were five clinical procedures (nasogastric tube insertion; urinary catheter insertion; preparation, reconstitution, dilution and administration of IV drugs; blood sampling and blood cultures from central line and tunnelled line; and sterile field set-up) that, even by the third time point, the majority of the interns had not carried out in the past week. This information should be considered in terms of how many repetitions are required to become competent in a procedure. For example, it was found that it took junior doctors between 19 and 146 repetitions of peripheral venous cannulation procedures in the clinical environment to reach an acceptable level of performance ^21^. Therefore, despite their confidence in their abilities, it may be that the intern are not receiving sufficient exposure to these clinical procedures in order to reach competency. Particularly for procedures for which the interns receive limited exposure, there is a need to give additional opportunities for them to practice and receive feedback on these skills. It is suggested that simulation-based education provides a mechanism for interns to become competent, in a safe learning environment.

## Recommendations

The Irish intern year EPAs provide a framework for medical schools to design teaching and assessment to better prepare newly graduated medical students for internship and clinical practice. A higher level of preparedness of medical students at the beginning of internship would have a number of benefits. The newly graduated students could be formally assessed to ensure they are competent to start internship, it would reduce the need for ‘boot camps’and on-the-job learning of these EPAs. Given that most learning occurred during the first three months of training, it is suggested that extra support should be available for interns during this time period. A particular approach used in the ITN that was the subject of our research is a ‘buddy’ intern programme. In this programme, ‘buddy’ interns are recruited from a pool of intern volunteers who have just completed the intern year. The ‘buddy’ interns work alongside the new interns for the first month and provide support and mentoring, skills training, and work night shifts. There was strong support for this programme from the participating newly graduated doctors ^22^.

There is a need to assess entrustability, beyond self-report. It has been suggested that there is general tendency for doctors to be over confident in their ability to complete particular clinical tasks ^23^. Therefore, we cannot draw conclusions about actual levels of entrustability as the interns’ competence has not been formally assessed. Such assessment will allow the identification of those interns that are not able to perform these without direct supervision, and provide them with extra training required. Approaches to the assessment of EPAs that have particular potential include the use of simulation and portfolios ^6, 24, 25^. However, there is a need for research on how such tools can be used- to include a consideration of the feasibility of these assessments being carried out by busy clinical supervisors ^10^.

### Limitations

The main limitation of the research reported in this assignment is that the data is based on self-report. As discussed above, there may be a tendency for the interns to be over-confident in the ability to execute the EPAs. So, these findings should be considered with that in mind. There are also a number of other limitations that should be acknowledged. The research was carried out during the COVID-19 pandemic. Therefore, the findings may not be the same as if the data was collected during a more typical intern training year. The response rate although high at baseline, dropped considerable on each occasion that the survey was distributed- corresponding with a time in which there was a COVID-19 surge in Ireland. Finally, only respondents from one Irish ITN were surveyed.

## Conclusion

After three months of clinical practice, almost all of the interns believed they could execute all of the EPAs in the Irish intern EPA framework without the need for direct supervision. This is certainly a positive finding. However, consideration should be given as to how to better prepare medical students to execute these EPAs as an integral part of their undergraduate training, how the interns can be better supported during the first quarter of internship, and there is an urgent need to carry out formal assessments of entrustability, rather than relying on self-report. Adopting this type of competency based approach will benefit the interns (as they will know they have the competencies required to perform the job), their supervisors (who know they can trust the interns), and most importantly their patients- the main beneficiaries of well-trained interns.

## Data availability

### Underlying data

Zenodo: An evaluation of self-reported level of entrustment across the first year of clinical practice. https://doi.org/10.5281/zenodo.5825638 ^16^.

This project contains the following underlying data:

□ Survey responses time 1: EPA survey time 1.sav
□ Survey responses time 2: EPA survey time 2.sav
□ Survey responses time 3: EPA survey time 3.sav
□ Survey responses time 4: EPA survey time 4.sav

### Extended data

Zenodo: An evaluation of self-reported level of entrustment across the first year of clinical practice. https://doi.org/10.5281/zenodo.5825638.

This project contains the following extended data:

□ Survey A: Questionnaire time 1.docx
□ Survey B: Questionnaire time 2–4.docx

Data are available under the terms of the Creative Commons Attribution 4.0 International license (CC-BY 4.0).
