# Supplementary material for: Longitudinal survey of self-reported level of entrustment across the first year of clinical practice
Source: HRB Open Res. 2022 Jan 20;5:9. [Version 1] doi: 10.12688/hrbopenres.13487.1 (PMC9005985; doi:10.12688/hrbopenres.13487.1)
Supplement: Peer review history

# Peer review: Reviewer response for version 1

**DOI:** 10.21956/hrbopenres.14708.r31756

**Published:** 2022-04-12

**Authors:** Christoph S. Burkhart

**Affiliations:** 1 Kantonsspital Graubunden, Chur, Switzerland

The authors analysed self-reported levels of entrustment in interns in their first year of clinical practice in one national intern training network in Ireland. Interns were asked to self-assess their performance in the seven Entrustable Professional Activities (EPAs) in the EPA framework for interns in Ireland (clerk a patient, request and interpret basic investigations, perform essential procedural skills, manage the work of in-patient care, Prescribe and monitor drugs and fluid, recognize and manage the deteriorating/acutely unwell patient and handover and discharge a patient.)

 They found that a high percentage of participants feel confident to execute the Irish first year EPAs with either reactive supervision or supervision at a distance after only 3 months of training. The authors conclude that internship is effective in increasing the level of competence of junior doctors. This is an important finding, however, the database are self-reported and may be prone to bias ^1^. It may be important to confirm the results of these self-assessments by assessments of supervisors.

Is the work clearly and accurately presented and does it cite the current literature?

Yes

If applicable, is the statistical analysis and its interpretation appropriate?

Not applicable

Are all the source data underlying the results available to ensure full reproducibility?

Yes

Is the study design appropriate and is the work technically sound?

Partly

Are the conclusions drawn adequately supported by the results?

Partly

Are sufficient details of methods and analysis provided to allow replication by others?

Yes

Reviewer Expertise:

EPAs

I confirm that I have read this submission and believe that I have an appropriate level of expertise to confirm that it is of an acceptable scientific standard.

## References

[1] : Unskilled and unaware of it: how difficulties in recognizing one's own incompetence lead to inflated self-assessments. *J Pers Soc Psychol* .1999;77(6) : 10.1037//0022-3514.77.6.1121 1121-34 10.1037//0022-3514.77.6.1121 10626367

# Peer review: Reviewer response for version 1

**DOI:** 10.21956/hrbopenres.14708.r31303

**Published:** 2022-02-08

**Authors:** Terri P. McVeigh

**Affiliations:** 1 Cancer Genetics Unit, Royal Marsden NHS Foundation Trust, London, UK

The authors have outlined a study in which interns in a single Irish intern training network were surveyed at 4 time points over the course of their intern year and asked to self-assess their entrustability in each of the 7 Entrustable Professional Activities (EPAs) outlined in the EPA framework for Irish interns - as well as providing specific entrustability ratings for 12 procedural skills under EPA3. Participants were also asked to comment on the frequency at which they performed these skills in clinical practice. 

 The authors demonstrate a moderate correlation between self-assessed entrustability and the frequency at which the EPAs were performed. 

 Somewhat surprisingly, some of the core tasks (urinary catheterisation, were performed weekly by only a minority of interns, even by the last time point, IV drug administration, NG insertion, etc), and yet median self-reported entrustability for all such tasks was 4.  As the authors point out, confidence does not necessarily correspond to competence, and those respondents rating their skills highly without sufficient practice may be unaware of their developmental needs in this area. 

 The attrition of respondents across the four time points is also a significant limitation of the study, with just over a quarter of potential participants responding to the final survey - despite the best efforts of the authors to increase participation with the addition of a prize draw for later surveys. This attrition limits the strength of conclusions that can be drawn from this study - it is possible that those participants who continued to respond are biased towards the more confident. 

 The survey also collected some basic demographic data including age, gender, and whether training was in Ireland or in another country, but no analyses including these data were undertaken. Response rates in the group <24 reduced over time, while those in the group 25-34 increased - I wonder if this reflects "aging up" of a number of respondents into the next category rather than discordant trends across age groups. 

 An important finding from this study is the low rate of self-reported entrustability in core skills at the end of undergraduate medical training, in line with previous work showing that interns feel underprepared for clinical practice after medical training. The authors have suggested useful strategies to address this and support these medics in the transition from student to doctor.  

 Ultimately, entrustment judgements should be informed by a number of pieces of evidence.  It would be interesting to determine if the self-assessment of the participants corresponded with the judgements of their supervisors or peers.

Is the work clearly and accurately presented and does it cite the current literature?

Yes

If applicable, is the statistical analysis and its interpretation appropriate?

Yes

Are all the source data underlying the results available to ensure full reproducibility?

Yes

Is the study design appropriate and is the work technically sound?

Yes

Are the conclusions drawn adequately supported by the results?

Yes

Are sufficient details of methods and analysis provided to allow replication by others?

Yes

Reviewer Expertise:

Medical Education (Assessment), Clinical Genetics, Cancer

I confirm that I have read this submission and believe that I have an appropriate level of expertise to confirm that it is of an acceptable scientific standard.
